# Comparative genomics and metabolomics reveal phytohormone production, nutrient acquisition, and osmotic stress tolerance in *Azotobacter chroococcum* W5

**DOI:** 10.3389/fmicb.2025.1626016

**Published:** 2025-07-22

**Authors:** M. Elakkya, Luz A. González-Salazar, Karina López-Reyes, Inês Rebelo-Romão, André Sousa, Victoria Gödde, Karsten Niehaus, Dhivya P. Thenappan, Juan Ignacio Vilchez, Sangeeta Paul, Cuauhtemoc Licona-Cassani

**Affiliations:** ^1^Division of Microbiology, ICAR-Indian Agricultural Research Institute, New Delhi, India; ^2^Research and Development Division, Sea6 Energy Pvt Ltd., C-CAMP, NCBS-TIFR, Bengaluru, India; ^3^Industrial Genomics Laboratory, Centro de Biotecnología FEMSA, Escuela de Ingeniería y Ciencias, Tecnologico de Monterrey, Monterrey, Mexico; ^4^Instituto de Tecnologia Química e Biológica (ITQB)-NOVA, iPlantMicro Lab, Oeiras, Lisbon, Portugal; ^5^Proteom-und Metabolomforschung, Fakultät für Biologie, Centrum für Biotechnologie, Universität Bielefeld, Bielefeld, Germany; ^6^Systems Plant Physiology, Texas A&M AgriLife Research and Extension Center, Uvalde, TX, United States; ^7^Tecnológico de Monterrey, The Institute for Obesity Research, Monterrey, Mexico; ^8^Instituto de Biotecnología, Universidad Nacional Autónoma de México, Cuernavaca, México

**Keywords:** Azotobacter chroococcum, comparative genomic analysis, pan-genome, plant growth-promoting rhizobacteria, seed germination, wheat

## Abstract

**Introduction:**

Concerns about ecological degradation and reduced biodiversity have intensified the search for sustainable solutions in agriculture. The use of plant growth-promoting bacteria (PGPB) offers a promising alternative to enhance soil quality and crop yield while reducing the consumption of chemical fertilizers.

**Methods:**

Here, we characterize the plant growth-promoting potential of *Azotobacter chroococcum* W5 through comparative genomics, in vitro experiments, and metabolomic analyses.

**Results:**

Comparative genomic analysis revealed plant growth-promoting traits, including phytohormone biosynthesis, nutrient acquisition, stress adaptation, and colonization in the *A. chroococcum* W5 strain. Experimental assays confirmed the production of auxin, gibberellic acid, phosphate solubilization, moderate nitrogen fixation, and growth on ACC. Wheat seed inoculation significantly enhanced germination metrics, seedling vigor, and altered carbohydrate metabolism in the seed endosperm. Under salt and osmotic stress, *A. chroococcum* W5 metabolomic profiling revealed adaptive responses, including elevated levels of osmoprotectants (proline, glycerol) and oxidative stress markers such as 2-hydroxyglutarate, while putrescine and glycine decreased.

**Discussion:**

Our results show that the *A. chroococcum* W5 strain has great potential for the development of novel formulations. More importantly, our results highlight the potential of using plant growth-promoting microorganisms for innovative, sustainable solutions in agriculture.

## 1 Introduction

Many regions have turned to intensified agricultural practices such as monocropping, excessive use of chemical pesticides and synthetic fertilizers to meet the food demand projected by 2050 (Viana et al., 2022; Agbodjato and Babalola, 2024). These practices promote soil degradation, contaminated water sources, and significantly reduced biodiversity, posing long-term risks to ecosystem resilience (de Andrade et al., 2023). Sustainable agricultural practices have emerged as potential solutions to mitigate environmental degradation and ensure food security (Calicioglu et al., 2019). The use of plant-associated microorganisms significantly promotes plant health and productivity by facilitating nutrient acquisition, enhancing stress tolerance, and modulating plant immune responses (Bashan and de-Bashan, 2010).

*Azotobacter chroococcum*, a free-living, obligate aerobic diazotroph from the class Gammaproteobacteria, is ubiquitous in diverse soil ecosystems and has been extensively studied for plant growth-promoting (PGP) applications (Viscardi et al., 2016). In addition to nitrogen fixation, *A. chroococcum* exhibits phytohormone production, phosphate solubilization, siderophore synthesis, and the release of antifungal metabolites, conferring biocontrol potential against phytopathogens (Mrkovački and Milić, 2001). The positive effects of *A. chroococcum* inoculation on plant growth and productivity have been noted in economically important field crops, such as cereals and pulses (Wani et al., 2013).

Studies on different isolates of *A. chroococcum* have shown an increase in nitrogen and phosphorus levels when used for seed inoculation of maize at different altitudes (Pandey et al., 1998) and under salinity conditions (Abdel Latef et al., 2020). Co-inoculation experiments involving *A. chroococcum* and the fungus *P. indica* have facilitated the acquisition of nitrogen and phosphorus in rice (Bandyopadhyay et al., 2022). *A. chroococcum* demonstrated their ability to help wheat plants tolerate drought conditions while improving grain yield and nitrogen, phosphorus, and potassium contents (Saad et al., 2020). Additionally, the inhibitory effect of *A. chroococcum* has been evaluated against plant pathogens such as *Aspergillus, Fusarium*, and *Alternaria* (Chahande, 2023). While the application of *Azotobacter* sp. is promising, in-depth genomic, metabolomic, and phenotypic characterization of individual strains is essential to design effective bioformulations for novel agricultural applications.

Here, we integrated comparative genomics and experimental data to explore the functional basis underlying plant growth-promoting traits of *A. chroococcum* W5 isolated from the rhizosphere of wheat grown in the fields of ICAR-Indian Agricultural Research Institute, New Delhi-110012, India (Kannepalli et al., 2020). In addition to genomics analysis, our *in vitro* experiments confirmed that *A. chroococcum* W5 produces phytohormones (auxin and gibberellic acid), fixes nitrogen efficiently, forms biofilms, and stimulates early wheat seedling development. This combined analysis provides new insights into the molecular and metabolic features of *A. chroococcum* W5. It also offers a deeper understanding of the functional diversity in this genus and reinforces its potential use as a bioinoculant for sustainable agriculture.

## 2 Materials and methods

### 2.1 Bioinformatics analysis and comparative genomics

DNA extraction and genome sequence of *Azotobacter chroococcum* W5 (MTCC 25045) were previously reported by our research group (NCBI BioProject PRJNA610299, BioSample SAMN14292004) (Kannepalli et al., 2020). A database of 22 *Azotobacter* genomes was generated using publicly available genomes from the National Center for Biotechnology Information (NCBI) up to September 2023 (Table 1). Our selection criteria included genome assembly quality parameters such as N50, total genome size, GC content, and number of contigs. The genomes were annotated using the Rapid Annotations of the Subsystems Technology (RAST) version 1.3.0 available at https://rast.nmpdr.org/ (Glass et al., 2010). Plant growth-promoting (PGP) traits were analyzed using the PLant-associated BActeria web resource (PLaBAse) version 1.2.0 available at https://plabase.cs.uni-tuebingen.de/pb/plabase.php (Patz et al., 2021). The PGP-related gene prediction was conducted through Plant Growth Promoting Traits Prediction (PGPTs-Pred), a module designed to detect genes associated with plant-beneficial functions such as pathogen suppression, abiotic stress tolerance, nutrient acquisition, and rhizosphere competence. The prediction of PGPTs was performed using blastp version 2.10.1 (Camacho et al., 2009) and HMMER version 3.3.0 (Finn et al., 2011) in strict mode with default parameters. The identified genes were further classified based on their functional categories and compared against a database of known PGP bacterial genomes (Patz et al., 2021). To avoid overestimation, the initial list of predicted genes was filtered to exclude housekeeping and highly conserved genes not specific to plant-associated functions (Bourguet and Guillemaud, 2016; Croning et al., 2009). Additionally, a subtractive analysis was performed using the genome annotation of the phytopathogen Pseudomonas syringae pv. syringae B728a (GCF_000012245.1).

**TABLE 1:** Genomic information about *Azotobacter* strains analyzed in this study.

Genome name	Isolation	GenBank assembly	No. of contigs	N_50_	Length (Mb)	G+C (mol %)	References
*Azotobacter beijerinckii* DSM 378	Soil	GCA_900110885.1	186	84.9	4.94	65.5	Fallik et al., 1991
*Azotobacter beijerinckii* DSM 373	Soil	GCA_900108885.1	249	43	5.1	65.5	Not found
*Azotobacter beijerinckii* DSM 1041	No reported	GCA_900108965.1	242	66.1	5.1	65.5	Not found
*Azotobacter beijerinckii* DSM 282	Soil	GCA_900112015.1	245	43.5	4.9	65.5	Not found
*Azotobacter beijerinckii* DSM 381	Soil	GCA_900114395.1	261	42.7	4.9	65.5	Not found
*Azotobacter chroococcum* B3	Soil	GCA_002220155.1	4	4.6	5	66	Fallik et al., 1991
*Azotobacter chroococcum* P207	Soil	GCA_004327885.1	69	224.5	4.6	66.5	Jin et al., 2020
*Azotobacter chroococcum* ATCC 9043	Soil	GCA_004327905.1	120	128.5	4.9	66	Jin et al., 2020
*Azotobacter chroococcum* P204	Soil	GCA_004327955.1	105	106.8	4.9	66	Jin et al., 2020
*Azotobacter chroococcum* DSM 2286	Wastewater	GCA_004339665.1	103	128.4	4.9	66	Jin et al., 2020
*Azotobacter chroococcum* P208	Soil	GCA_005144545.1	247	49.2	5.2	65.5	Fallik et al., 1991
*Azotobacter chroococcum* W5	Rhizosphere of wheat	GCA_011470035.1	55	258	4.6	66.5	Kannepalli et al., 2020
*Azotobacter chroococcum* hr1	Soil	GCA_016406165.1	5	4.6	5.1	66	Li et al., 2021
*Azotobacter chroococcum* NCIMB 8003	Soil	GCA_000817975.1	7	4.6	5.2	65.5	Gangoiti et al., 2016
*Azotobacter chroococcum* subsp. isscasi P205	Paddy soil	GCA_004327895.1	56	186	4.6	66.5	Jin et al., 2020
*Azotobacter salinestris* KACC 13899	Soil	GCA_009363155.1	3	4.9	5.3	65.5	Page and Shivprasad, 1991
*Azotobacter vinelandii* VKM B-1617	No reported	GCF_027922225.1	154	89.8	5.2	65.5	Steensma et al., 1996
*Azotobacter vinelandii* DSM 279	No reported	GCA_900119555.1	178	79.5	5.5	65	Trujillo-Roldán et al., 2003
*Azotobacter vinelandii* CA	Mutant strain	GCA_000380335.1	1	5.4	5.4	65.5	Noar and Bruno-Bárcena, 2013
*Azotobacter vinelandii* CA6	Mutant strain	GCA_000380365.1	1	5.3	5.3	65.5	Noar and Bruno-Bárcena, 2013
*Azotobacter vinelandii* DJ ATCC BAA-1	Soil	GCA_000021045.1	1	5.4	5.4	65.5	Noar and Bruno-Bárcena, 2013
*Azotobacter vinelandii* NBRC 13581	Soil	GCA_001571105.1	313	44	5.2	65.5	Shuvro et al., 2022

Pan-genome analysis was performed using the Bacterial Pan-Genome Analysis (BPGA) tool version 1.3.0 (Chaudhari et al., 2016), which uses the USEARCH algorithm version 11.0.0 (Edgar, 2010) to define orthologous genes. We set a strict similarity threshold of 70% to define shared genes across genomes. The protein sequences of core genes acquired through BPGA were used to construct a phylogenetic tree. The sequence alignments were concatenated with FasConCat version 1.11.0 (Kück and Longo, 2014), with default parameters, and IQtree software version 1.6.12 with default parameters was used for phylogeny construction (Nguyen et al., 2015). The phylogenetic tree was visualized using iTOL version 5.0.0 (Letunic and Bork, 2024).

Prediction of biosynthetic gene clusters (BGCs) was performed using the Antibiotic and Secondary Metabolite Analysis Shell (antiSMASH, version 7.1.0) with default parameters (Blin et al., 2017). BiG-FAM platform available at https://bigfam.bioinformatics.nl was used for comparative genomics to identify potentially novel BGCs (Kautsar et al., 2021). For BGC diversity, we used the Biosynthetic Gene Similarity Clustering and Prospecting Engine (BiG-SCAPE) platform, version 1.0.0 (Navarro-Muñoz et al., 2020). BiG-SCAPE scores were used to group BGCs into gene cluster families (GCFs), which were visualized as networks where nodes represent BGCs, and edges indicate similarity above a defined threshold (default: 0.3). Clusters that lack sufficient similarity to known types or do not present hallmark biosynthetic domains are categorized under the “Others” class (Navarro-Muñoz et al., 2020).

### 2.2 Quantification of PGP phenotypes of *Azotobacter chroococcum* W5

Auxin production was measured using the Glickmann method (Glickmann and Dessaux, 1995), determining the OD_530_. Each well added a calculated volume of 2 μL of bacterial culture to 200 μL of LB tryptophan-supplemented medium (0.5 g/L). After 48 h of incubation (28°C, 180 rpm), the 96-well plates were centrifuged (1,800 × g 50 min) in the Eppendorf 5810R centrifuge. Following centrifugation, 100 μl of Salkowski’s reagent (For 50 mL solution: 1 mL (v/v) of 0.5 M FeCl_3_ to 49 mL of 35% HClO_4_) was added to 100 μL of the supernatant collected in fresh 96-well plates. Auxin levels were quantified using a calibration curve based on indole-acetic acid standards and expressed as μg/mL. Experiments were performed in five replicates using as control uninoculated media.

Gibberellic acid (GA) was determined using the modified Holbrook protocol (Berríos et al., 2004; Holbrook et al., 1961). *A. chroococcum* W5 culture was grown in Jensens’s N-free broth (28°C). After 5 days of incubation, 1 mL of the culture was centrifuged (10,000 × g, 10 min). The protein content of the cell lysate was estimated with Lowry’s method using a UV-visible spectrophotometer (Perkin Elmer, model Lambda EZ 201) (Berríos et al., 2004). GA production was expressed as μg GA/mg protein. Experiments were performed in three replicates using as control uninoculated media.

Nitrogenase activity was determined using the acetylene reduction assay (ARA) with slight modifications (Orozco-Mosqueda et al., 2020). Briefly, bacterial cultures were grown on Jensen’s agar slants (28°C, 72 h). For this experiment, we used test tubes with sterile suba seals. We replaced 10% of the air with acetylene gas and incubated for 24 h. After incubation, ethylene was quantified after injection of 1 mL of the air sample into a Gas Chromatograph (Nucon 5765) equipped with a flame ionization detector (FID), and a Porapak N column. Injector: Column: Detector was maintained at temperatures −80°C: 110°C: 110°C, respectively. Nitrogen was the carrier gas at a 30 mL/min flow rate. Three replicates, each inoculated and uninoculated (blank), were maintained. Protein content was determined from harvested rhizobacterial cultures. ARA was expressed regarding nmoles of ethylene produced/mg protein/h.

ACC deaminase activity was quantified by measuring OD_600_ using the Thermo Scientific Multiskan SkyHigh Microplate Spectrophotometer (Hageman and Hucklesby, 1971). A calculated volume of *A. chroococcum* W5 culture and 200 μL of M9 minimal medium was supplemented with 3 mM ACC in each well. M9 minimal medium was prepared, per liter: 100 mL of 10 × M9 salt solution; 1 mL of 1 M MgSO_4_; 0.3 mL of 1 M CaCl_2_; 10 mL of 100 x trace elements solution (TES). The M9 salt solution was prepared with 33.7 mM Na_2_HPO_4_, 22 mM KH_2_PO_4_, and 9.35 mM NH_4_Cl. The TES was prepared with 13.4 mM EDTA, 3.1 mM FeCl_3_-6H_2_O, 0.62 mM ZnCl_2_, 76 μM CuCl_2_-2H_2_O, 42 μM CoCl_2_-2H_2_O, 162 μM H_3_BO_3,_ and 8.1 μM MnCl_2_-4H_2_O. After incubating the 96-well plates for 72 h (28°C, 180 rpm), OD_600_ was determined using the Thermo Scientific Multiskan SkyHigh Microplate Spectrophotometer. Experiments were performed in three replicates using as control uninoculated media.

Bacterial biofilm production was determined using the Crystal Violet assay following the Coffey & Anderson method with minor modifications (Coffey and Anderson, 2014). Experiments were performed in 96-well plates containing the calculated volume of *A. chroococcum* W5 in LB medium and incubated for 48 h (28°C, 180 rpm). After incubation, plates were washed, and the biofilm remaining was stained with 200 μL of 0.5% crystal violet. After 10 min we rinsed with tap water to remove excess crystal violet and dried overnight. Then, 200 μL of glacial acetic acid 30% was added and plates were incubated for 10 min. OD_550_ was determined with Thermo Scientific Multiskan, SkyHigh Microplate Spectrophotometer. Three independent experiments were performed with their respective controls.

### 2.3 *In-vitro* assays on wheat seeds

Two wheat varieties (*Triticum aestivum*, HD2967 and *Triticum durum*, HI8759) were obtained from the Division of Genetics, ICAR- Indian Agricultural Research Institute, and the Regional Station, Indore, Madhya Pradesh, respectively. Three treatments with three independent replicates were used for seed inoculation: (1) rhizobacterial culture broth (48 h old cultures, OD = 1.0), (2) cell-free extract containing cell lysate obtained by sonication, and (3) cell culture supernatant. Cell-free extracts were obtained from pellets (10,000 rpm, 10 min) generated from 2 mL bacterial cultures (OD = 1) in Jensen broth. The cell pellets were washed twice with phosphate-buffered saline (pH = 7.0) and then 1 mL of extraction buffer (50 mM Tris (Hydroxymethyl) aminomethane (pH = 8.3), 0.5 M Sucrose, 50 mM EDTA-Na, 2 mM PMSF, 0.1% (v/v) 2-mercaptoethanol). After sonication for 10 min (12.5% pulse cycle, 30 s mode on,10 s mode off), a suitable aliquot was plated on Jensen’s agar to verify the absence of cell growth. Bacterial supernatants were obtained from 2-day-old liquid cultures (OD = 1), centrifuged (10,000 x g,10 min), and filter sterilized with a 0.45 μm syringe filter. A suitable aliquot was spread plated on Jensen’s agar plates to verify the absence of bacterial growth.

### 2.4 Germination assessment

Surface-sterilized wheat seeds (*T. aestivum* and *T. durum* varieties) were treated with 0.5 mL of the *A. chroococcum* W5 suspension. Treatments included rhizobacterial broth (48 h-cultures, OD = 1.0), cell-free lysates, culture supernatants, and sterile water as a control. The seeds for each treatment were incubated for 30 min and then transferred to 0.8% agar plates and incubated in a BOD incubator at 25°C for 24 – 72 h (Roessner et al., 2000). Seedling germination percentage, seedling vigor index, fresh weight, plumule, and radical length were measured on the first, second, and third days as previously reported (Nivetha et al., 2024). The seed germination percentage was calculated based on the number of seeds germinated about the total number of seeds. The seedling vigor index was calculated using the following formula:


Seedlingvigorindex(SVI)=GerminationPercentage×

M⁢e⁢a⁢n⁢S⁢e⁢e⁢d⁢l⁢i⁢n⁢g⁢L⁢e⁢n⁢g⁢t⁢h⁢(c⁢m)

### 2.5 Biochemical and enzymatic assays

Biochemical activity in the seed endosperm and N assimilating enzymatic activity for seedlings were assayed on the first, second, and third days of germination. Starch content was quantified following the Anthrone method (Wang et al., 2014). The glucose concentration was multiplied by 0.9 to estimate the starch yield. Amylose content was measured using the iodine binding method at OD_625_ using a standard curve (Brust et al., 2020). Amylopectin content (%) was calculated by subtracting amylose content from the total starch content (General Carbohydrate Method, 1972). Three replicates for each treatment were performed.

Seedlings (1 g) were assayed for Glutamine synthetase (GS, EC 6.3.1.2), Glutamate synthase (GOGAT, EC 1.4.1.14), and Glutamate dehydrogenase (GDH, EC 1.4.1.2) (Mohanty and Fletcher, 1980). Fresh seedlings were homogenized in a 100 mM Tris-HCl buffer (100 mM Tris-HCl, 100 mM sucrose, 10 mM EDTA, and 10 mM MgCl_2_) using a pre-cooled pestle and mortar. The homogenate was centrifuged (5,000 × g, 10 min, 4°C), and the resulting supernatant was centrifuged (12,000 × g, 15 min, 4°C). The cell lysate obtained was used for the GDH assay, and the supernatant for the GS and GOGAT assays.

GS activity was quantified by incubating the reaction mixture at 37°C for 30 min. The reaction mixture contained 0.35 mL of 200 mM Tris buffer, 0.25 mL of 200 mM MgSO_4_, 0.1 mL of 50 mM cysteine, 0.25 mL of 500 mM α-glutamate, 0.1 mL of 50 mM ATP, 0.25 mL of 40 mM hydroxylamine, and 0.2 mL of crude enzyme extract. We added 0.5 mL of FeCl_3_ reagent (10 g FeCl_3_, 3.25 g trichloroacetic acid, 5.92 mL HCl in 100 mL ddH_2_O) to stop the reaction, followed by centrifugation at 1,000 × g for 10 min. The enzyme activity was measured as γ-glutamyl hydroxamate production/g fresh weight/h, with absorbance determined using a UV-visible spectrophotometer. Three replicates for each treatment were performed.

GOGAT activity was determined with a reaction mixture containing 1 mL of 75 mM Tris-HCl buffer, 0.2 mL of 50 mM α-ketoglutaric acid, 0.2 mL of 200 mM L-glutamine, and 0.1 mL of crude enzyme extract, and the volume was made up to 2.8 mL. We added 0.2 mL of 1.5 mM NADH, and absorbance was measured immediately for 60 s at 340 nm, using a UV-visible spectrophotometer. The enzyme activity was measured as μmol NADH oxidized/g fresh weight/h. Three replicates for each treatment were performed.

GDH activity was measured by resuspending the cell lysate in a 50 mM phosphate buffer (pH = 7.5) containing sucrose (0.0625 M). The reaction mixture contained 1 mL of 75 mM phosphate buffer, 0.2 mL of 100 mM α-ketoglutaric acid, 0.4 mL of 750 mM NH_4_Cl, and 0.2 mL of crude enzyme extract. We added 0.1 mL of 1.5 mM NADH and measured absorbance for 60 s at 340 nm. Enzyme activity was measured as μmol NADH oxidized/g fresh weight/h. Three replicates for each treatment were performed.

Nitrate reductase activity (NR, EC 1.7.1.15) was quantified from seedlings (0.5 g) as previously reported (Hageman and Hucklesby, 1971). Fresh seeds were homogenized using a chilled pestle and mortar with 1 mL of cold extraction buffer (0.1 M phosphate buffer, pH = 7.5, containing 5 mM EDTA and 5 mM cysteine). We added 0.5 mL of the enzyme extract to the reaction mixture containing 1.9 mL of phosphate buffer (0.1 M, pH = 7.5), 500 μl of 0.1 M KNO_3_, and 100 μl of 10 mM NADH. After 30 min, the reaction mixture was stopped using 0.2 mL of 1 M zinc acetate and 1.8 mL of ethanol 75%. The reaction mixture was centrifuged (400 × g, 5 min) and added 1 mL sulfanilamide solution 1% (w/v) and 1 mL N-(1-naphthyl) ethylene diamine solution of 0.02% (w/v). After 20 min, enzyme activity was measured (540 nm) and expressed as μmol nitrite formed/g fresh weight/h. Three replicates for each treatment were performed.

Phytohormone Gibberellic acid (GA) content in 3-day old seedlings was quantified according to Bhalla et al. (2010). Germinated seedlings (2 g) were homogenized in liquid nitrogen. We added 10 mL sodium phosphate buffer (50 mM, pH = 7.5) with 0.02% sodium diethyl dithiocarbamate and incubated the extracts overnight at 4*^o^*C, 150 rpm. After centrifugation (10,000 × g, 10 min, 4*^o^*C), the supernatants were collected and adjusted to 10 mL with sodium phosphate buffer. Then, we added 5 mL of diethyl ether for partitioning using a separating funnel. The lower aqueous phase was separated and acidified to pH = 2.5 using 1 N HCl, followed by partitioning twice with 5 mL of petroleum ether. The aqueous phase was combined and further partitioned twice using diethyl ether. The collected aqueous phase was combined and partitioned twice with 2.5 mL of ethyl acetate. The upper organic layers were collected, combined, and partitioned twice with 5 mL of 0.2M K_2_HPO_4,_ followed by a collection of the aqueous layer. After adjusting the pH to 2.5 with concentrated H_3_PO_4_, the mixture was partitioned twice with 5 mL ethyl acetate. The upper organic layer was collected and filtered through anhydrous sodium sulfate crystals. After filtration, the ethyl acetate was evaporated using a rotary evaporator. The residue obtained after drying was dissolved in 1 mL of absolute methanol (HPLC grade), followed by filtering through syringe filters (0.45 μm). The chromatographic separation was performed using an Agilent 1,200 series HPLC system with a UV-visible detector set at 206 nm. The mobile phase (methanol:water; 60:35), was used at a flow rate of 1.5 mL/min through a LiCHrospheron RP-18 column. Data acquisition and analysis were conducted using ChemStation software. Endogenous GA content was quantified based on a standard curve and expressed as μg/g fresh weight, with three replicates for each treatment.

### 2.6 Metabolite extraction

*A. chroococcum* W5 was inoculated into 250 mL Luria Bertani (LB) broth (OD = 0.1). Treatments included stress PEG 8,000 (15 and 30%), salt (90 mM and 180 mM as NaCl), and untreated (as control). Three replications of each treatment were maintained. Supernatants and cell pellets generated from bacterial cultures (centrifuged at 10,000 x g, 30 min) were freeze-dried and stored at −80°C. To evaluate the contribution of distinct metabolic pools, we analyzed both the cell culture supernatant and the cell-free extract containing the lysate of disrupted cells. The rationale behind using these two fractions was to differentiate between metabolites secreted into the medium during active growth (supernatant) and intracellular compounds released upon cell lysis (lysate), such as enzymes and secondary metabolites. The samples were homogenized using micropistils in 1.5 mL (Roessner et al., 2000). Metabolites were extracted from 5 mg of sample material using 1 mL of 80% methanol (with 10 μM ribitol as internal standard). The extraction was performed using a Precellys24 Instrument (Peqlab, Erlangen, Germany) with 1 mm zirconia beads (Roth, Karlsruhe, Germany) for three-45 s cycles. Following centrifugation (15,000 x g, 20 min), 750 μl of the clear supernatant was transferred to 1 mL glass vials (Supelco, Bellfonte, California) and evaporated under a nitrogen stream. The dried metabolites were derivatized by adding 75 μL of methoxylamine hydrochloride in pyridine (20 mg/mL) and incubating for 90 min at 37°C, followed by the addition of 75 μl of N-Methyl-N-(trimethylsilyl) trifluoroacetamide (MSTFA) and incubation for 30 min at 37°C. All chemicals and standard compounds were sourced from Sigma-Aldrich-Fluka (Taufkirchen, Germany), Merck (Darmstadt, Germany), or Macherey-Nagel (Düren, Germany).

### 2.7 GC-MS analysis

For the GC-MS analysis, 1 μL of the derivatized sample was injected into a TSQ 9,000 Triple Quadrupole GC-MS/MS system (Thermo Electron, Dreieich, Germany). The metabolites were vaporized at 280°C in split-less mode and separated on a 30 m x 0.25 mm OPTIMA-5MS capillary column with a 0.25 μm film thickness. Helium was used as the carrier gas at a 1 mL/min flow rate. The interface temperature was maintained at 250°C, and the ion source temperature at 280°C. The oven temperature was initially held at 80°C for 3 min, then increased to 320°C at a rate of 5°C/min and held at 320°C for 2 min. Post-analysis, the system was equilibrated at 80°C for 2 min. Mass spectra were acquired at 1 scan per second over a mass range of 50–750 *m/z*. Metabolites were identified by comparing the spectra to purified standards and the Golm Metabolome Database (version 2022). The relative levels of the selected metabolites were quantified by integrating the peak areas of selective ions using the Xcalibur 4.5 software (Thermo Electron, Dreieich, Germany), with relative response ratios calculated by normalizing the peak areas to the internal standard and adjusting for the sample weight (Farajzadeh et al., 2014).

### 2.8 Statistical analysis

All data are presented as mean values with standard deviations for each treatment. Statistical analysis was performed using one-way ANOVA, followed by the LSD multiple range test (*P*< 0.05) with the GRAPES (General R shiny based Analysis Platform Empowered by Statistics for data analysis in Agriculture-part1) platform version 1.1.0 available at http://www.kaugrapes.com (Gopinath et al., 2021). We used Pearson’s correlation coefficient (r) to determine the strength and direction of the linear association between variables at the 5% significance level. The analyzed variables were quantitative and followed normal distribution, thereby justifying the use of Pearson’s method for analysis. For visualization, variables that showed significant correlation (*p*< 0.05) with bacterial growth were selected, while those without significant associations were excluded.

## 3 Results

### 3.1 Genomic features of *A. chroococcum* W5 specialized for plant growth-promoting functions

We analyzed the genome of *A. chroococcum* W5 using the Plant Growth-Promoting Traits (PGPTs) module from PLaBAse. This approach identified 1,643 PGPTs, including 988 related to direct effects and 655 associated with indirect effects. We found that 19.49% of PGPTs were related to nutrient acquisition, 10.29% to phytohormone plant signal production, and 10.11% to bioremediation (Supplementary Figure 1A). Within the genetic features related to indirect effects, we found that 29.96% of PGPTs were associated with colonizing plant systems, 19.31% with competitive exclusion, 10.78% with stress control and stress signaling proteins, and 0.06% were related to plant immune response stimulation (Supplementary Figure 1B).

We identified plant growth-promoting traits (PGPTs) related to nitrogen and phosphate assimilation, as well as potassium availability (Supplementary Table 1). For nutrient assimilation, we found genes encoding nitrogen uptake genes (*nas R, S, T*), genes involved in nitrogen-related enzymatic activities (*nosX)*, and dicarboxylate uptake (*dctA*). We also identified genes associated with phosphate storage and mobilization (*phoD, ppa, ppx*, and *ppk)*, and potassium transport (*kdpA-E* and *trk* systems).

Regarding antibiotic resistance and nutrient assimilation, we identified a gene encoding an outer membrane efflux protein (*oprM*). We also identified genes involved in polyamine biosynthesis, which support plant stress tolerance and growth (*speF, speC, ODC1*). For motility, we found regulators of flagellar biosynthesis (*flhD, flhC*). Additionally, we detected a gene encoding a component of a type I secretion system involved in detoxification (*tolC*), a sensor kinase associated with environmental stress adaptation (*pmrB*), and a gene participating in exopolysaccharide biosynthesis that facilitates biofilm formation and root surface adhesion (*rfbA*).

### 3.2 *A. chroococcum* W5 genome encodes for unique genes related to PGP functions within the *Azotobacter* pan-genome

**FIGURE 1:**
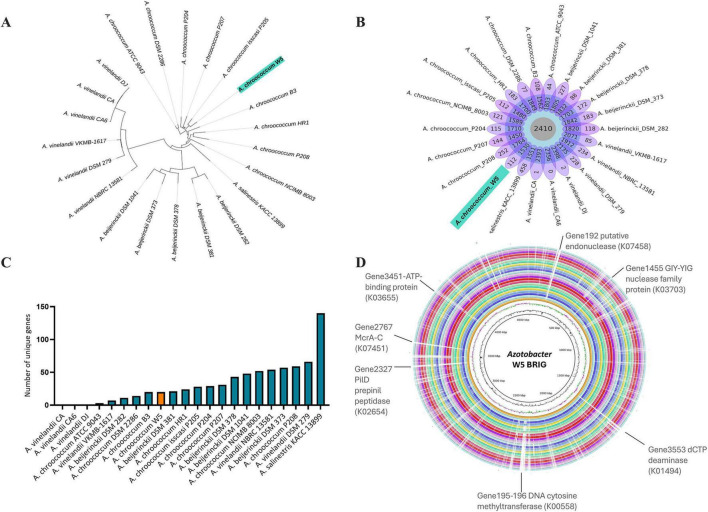
Pan-genome analysis for *Azotobacter* genomes. **(A)** Phylogeny tree based on the pan-matrix from BPGA analysis for the *Azotobacter* strains. The alignment was performed with muscle v.3.8.31. In red is highlighted the strain. **(B)** Flower diagrams represent each genus’s core, unique, and accessory genes. The numbers underneath the names represent the number of accessory genes. The pan-genome is open. **(C)**. Distribution of unique genes, KEGG classification of unique genes in *A. chroococcum* W5. **(D)**. Circular plots of Azotobacter. The three innermost rings indicate the genome size, GC content, and GC skew of the reference genomes *Azotobacter* sp. W5. The outer rings illustrate the BLAST genome comparisons against the genome reference. From the inside to the outside, the rings correspond to the following strains: W5, P205, P207, P204, 2286, 9043, B3, HR1, P208, 8003, 13899, 282, 381, 378, 373, 1041, 13581, 279, 1617, CA, CA6, DJ. The outermost ring represents unique genes and KEGG assignment.

To infer the phylogenetic relationships among *Azotobacter* strains, we reconstructed a core-genome phylogenetic tree (Figure 1A). The 22 analyzed strains clustered into three clades, with *A. chroococcum* W5 closely related to *A. chroococcum* P205 from paddy soil in China [48]. We did not observe correlations between phylogeny and isolation sources. The estimated *Azotobacter* pan-genome comprises 45,902 genes, with 2,410 core genes, an average of 1,830 accessory genes, and 3,064 unique genes (Figure 1B; Supplementary Table 2). In our analysis using BPGA, the resulting power-law function was Ps = 4,570.25⋅n^0.287^, where the exponent γ = 0.287 confirms an open pan-genome model (Supplementary Figure 2), highlighting the need for further strain isolation. Based on the BPGA assignation of unique genes clustered using USEARCH at 70% sequence identity, *A. vinelandii* (CA, CA6, DJ) harbors the fewest unique genes (0–2), whereas *A. beijerinckii* DSM 1041, *A. vinelandii* DSM 279, *A. vinelandii* NBRC 13581, *A. chroococcum* P208, and *A. salinestris* KACC 13899 encodes a range of 227–458 unique genes.

Of the 112 genes identified as unique in *A. chroococcum* W5 (Figure 1C), eight were mapped to known pathways based on KEGG annotations. Among these, we found proteins annotated as an ATP-binding protein (K03655), a GIY-YIG nuclease family protein (K03703), and a putative endonuclease (K07458), potentially involved in homologous recombination, nucleotide excision repair, and mismatch correction, respectively. We also found genes encoding for dCTP deaminase (K01494) related to nucleotide metabolism, and cytosine-5 DNA methyltransferase (K00558), suggesting a role in DNA methylation and epigenetic regulation. Defense-related functions were also identified, such as McrA-C (K07451), encoding for methylated cytosines. In addition, the detection of PilD (K02654), a prepilin peptidase required for type IV pilus assembly, suggests that W5 may retain structures involved in motility, adhesion, or biofilm formation (Figure 1D).

A manual review of the remaining genes using BLAST analysis revealed additional functions related to metabolic processes, defense against foreign DNA, transcriptional regulation, and cellular homeostasis. Regarding metabolic processes, we found genes encoding a glycoside hydrolase family 24 protein, implicated in carbohydrate degradation. Genes with regulatory or translational functions were also present, such as YggL encoding for a 50 S ribosome-binding protein involved in ribosomal assembly. A sodium:proton antiporter and an outer membrane porin (OprD family) were detected, potentially important for ion balance and nutrient exchange. Genes associated with structural or mobile elements were identified, including a tyrosine-type recombinase/integrase and a TadE/TadG family pilus assembly protein, suggesting potential roles in DNA integration and surface adhesion, respectively. A complete list of these genes is provided in Supplementary Table 3.

### 3.3 Biosynthetic gene cluster pan genome analysis of *A. chroococcum* W5 against bacterial and fungal pathogens

**FIGURE 2:**
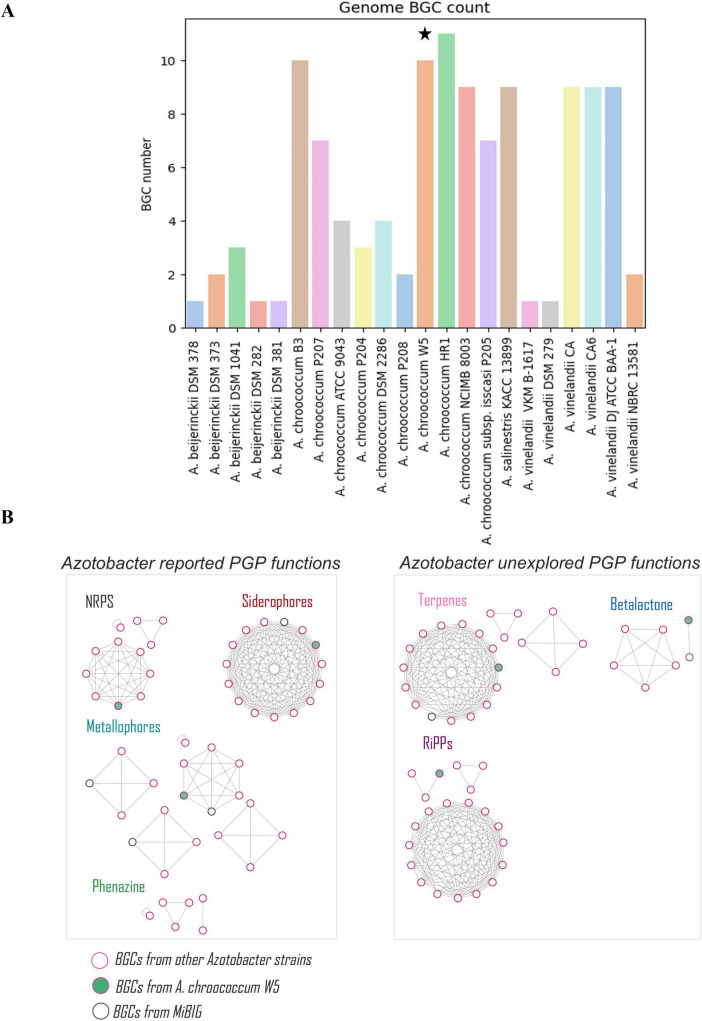
**(A).** Number of BGCs per *Azotobacter* strain. **(B)**. BGC network showing chemical diversity for *Azotobacter* strains, including chemical diversity related to PGP activities. The pink border color node corresponds to a BGC associated with an *Azotobacter* strain. The green color node represents *A. chroococcum* W5 BGCs. “*” denotes the focal strain used in this study (*Azotobacter chroococcum* W5).

Analysis of secondary metabolic pathways in *Azotobacter* identified 115 BGCs (mean = 5 per genome). Among these, 20% correspond to NRPSs, 12 % to PKSs, 7 % to RiPPs, 6 % to terpenes, and 4 % to hybrid PKS-NRPS. The majority of BGCs belong to other chemical families or remain unclassified (Supplementary Table 4). The *A. chroococcum* strains HR1, B3, and W5 have a higher number of BGCs (11, 10, and 10, respectively), while the *A. beijerinckii* strains DSM 378/282/381 and *A. vinelandii* strains VKM B-1617/DSM 279 have the fewest BGCs (Figure 2A). Comparative genomics of BGCs through homology networks revealed that *A. chroococcum* P204, *A. salinestris* KACC 13899, *A. chroococcum* NCIMB 8003, and *A. chroococcum* W5 (Supplementary Table 5) harbor the majority of unique BGCs (singletons) classified as NRPSs (Figure 2B).

### 3.4 Effect of *A. chroococcum* W5 plant growth-promoting properties on seedling germination

To validate the plant growth-promoting potential predicted from our genomic analyses, we quantified phytohormone production, evaluated nutrient assimilation traits, and assessed protective functions. *Azotobacter chroococcum* W5 produced 8.04 ± 0.97 μg/mg protein of gibberellic acid and 54.58 ± 8.46μg/mL of auxins after 48 h of incubation (Table 2). Auxin production was notably higher than values commonly reported for *A. chroococcum* strains. In contrast, the gibberellic acid concentration was within the range observed in previous studies; however, direct comparisons are limited due to variation in reporting units across the literature. Phosphate solubilization was low (0.074 ± 0.019 μg/mg protein) and, similarly, literature reports on this trait use diverse units, limiting direct quantitative comparison. Regarding nitrogen fixation, W5 showed acetylene reduction activity (ARA) of 4.51 ± 1.19 nmol ethylene/h/mg protein, a value within the reported range for this species, albeit near its lower limit. Biofilm formation was observed after 24 h (1.08 ± 0.14), and ACC deaminase activity reached 0.87 ± 0.005 after 72 h (Table 2).

**TABLE 2:** Values of phytohormones and PGP activities quantified in *A. chroococcum* W5.

PGP functional category	PARAMETERS	W5 STRAIN MEASUREMENTS	REFERENCE VALUES (REPORTED BY *AZOTOBACTER* STRAINS)
PLANT HORMONES	Gibberellic acid	8.038 ± 0.9682 (μg/mg protein)	0.01 and 0.1 μg/mL* (Sumbul et al., 2020)
	Auxins production (48h)	54.58 ± 8.464 (μg/mL)	42.80–82.00 μg/mL (Singh et al., 2014)
NUTRIENT ACQUISITION	Phosphate Solubilization	0.074 ± 0.0193 (μg/mg protein)	0.18 and 0.19 mg/L* (López Ortega et al., 2013)
NITROGEN FIXATION	Acetylene Reduction Assay	4.507 ± 1.1939 (n moles of ethylene/ h /mg protein)	1.31–846.56 n moles of ethylene/h/mg protein (Jain et al., 2021)
STRESS CONDITIONS	Growth in ACC (72 h)	0.87 ± 0.005 (O.D._600_)	N/A
BIOFILM FORMATION	Biofilm formation (24 h)	1.0845 ± 0.144 (O.D._550_)	N/A

*Direct comparison with literature values expressed in mg/L is limited due to differences in units and assay normalization. N/A, not applicable.

**FIGURE 3:**
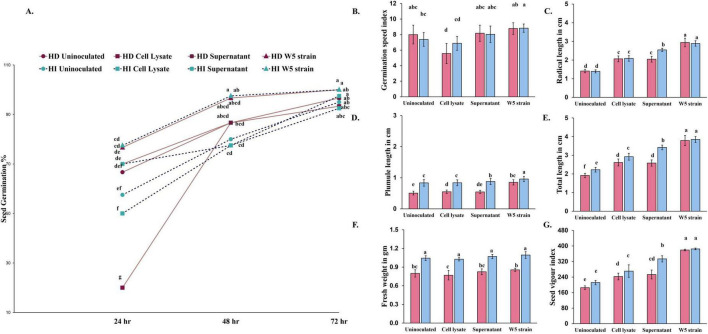
**(A)**. Germination percentage due to inoculation of W5 strain, and its components (Cell lysate and Supernatant) for two wheat genotypes, HD2967 & HI8759. **(B–G)** Column charts represent the physiological changes in the seedling. The different lowercase alphabets on the error bars indicate statistically significant differences between treatments (*p*≤ 0.05) performed by the Least significant difference test. Data are expressed as mean values with standard deviations for each treatment. Statistical analyses were performed using one-way ANOVA and an LSD multiple-range test with the GRAPES platform.

To demonstrate the growth promotion potential of *A. chroococcum* W5, we performed *in vitro* assays by inoculating wheat seedlings with live bacterial cultures, cell lysates, and cell culture supernatants (Figure 3A). For both varieties, seed germination was enhanced at 24–48 h only when live cells of W5 were inoculated onto seeds. This was also reflected in some of the parameters measured in seedlings, such as radicle and plumule length and seed vigor index, which showed stronger effects for live cells. The live bacterial suspension and cell lysate presented a significant increase in growth-promoting activity at 72 h. The germination speed index (GSI) showed a significant decline in wheat inoculated with cell lysate compared to the control (Figure 3B). After 72 h incubation with live bacterial cultures, we observed an increase in radical length (108.51, 107.32%), plumule length (68.24, 14.97%), total seedling length (97.86, 72.71%) and seedling vigor index (104.82, 81.89%) for both varieties HD2967 and HI8759, respectively (Figures 3C–E,G). Although the fresh weight increased with inoculation (7.13% for HD2967 and 4.79% for HI8759), this was non-significant (Figure 3F). Finally, while HI8759 variety treated with supernatant showed significant improvement (Figures 3C–E,G), HD2967 did not show significant improvement compared to the control. No significant changes in the fresh weight were observed (Figure 3F).

### 3.5 *Azotobacter chroococcum W5* induces biochemical changes in seed endosperm components

**FIGURE 4:**
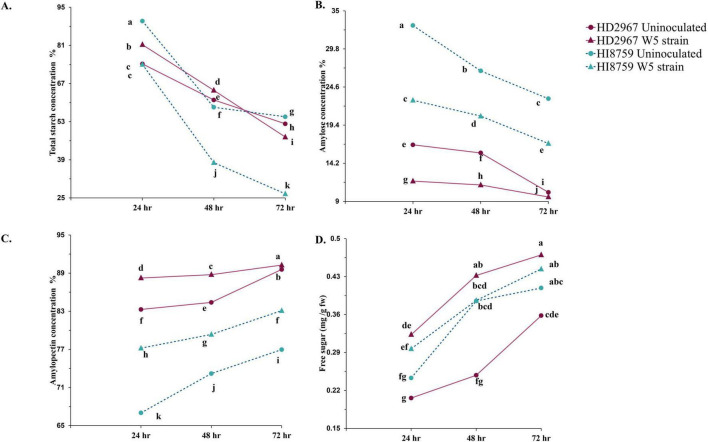
Temporal changes in the biochemical activities in the seed endosperm on 24, 48, and 72 h due to inoculation with W5 strain for two wheat genotypes, HD2967 & HI8759. **(A)** Total starch concentration. **(B)** Amylose concentration. **(C)** Amylopectin concentration. **(D)** Free sugar concentration. Different lowercase letters on error bars indicate statistically significant differences between the treatments (*p* < 0.05) performed by the Least significant difference test.

We evaluated different seed biochemical phenotypes, including total starch content, free sugars, amylose, and amylopectin concentrations in seed endosperm components (Figures 4A–D; Supplementary Table 6). We observed an overall decreasing trend in starch concentration across all treatments for both wheat varieties (Figure 4A). HI8759 showed significantly lower starch across the 72 h of incubation compared to the control. For HD2967, the inoculated treatment had a slightly higher starch content compared to the control for the first 48 h. By 72 h, both HD2967 and HI8759 showed significant declines in starch content compared to their respective controls. Similarly, amylose concentration decreased across all treatments over time (Figure 4B). However, control treatments for both wheat varieties maintained higher amylose content during the entire experiment.

In contrast, there was a significant increase in amylopectin for both varieties across all the treatments (Figure 4C). At 24 h, HI8759 showed a higher increase (77.12%) compared to uninoculated (67%). This pattern was observed at 48 h and 72 h, with increases of 79.34 and 83.10%, respectively, compared to the uninoculated treatment. For HD2967, we found lower increases at 24 h (88.24%), 48 h (88.76%), and 72 h (90.26%) compared to uninoculated treatments (83.28, 84.39, and 89.58%), respectively.

**FIGURE 5:**
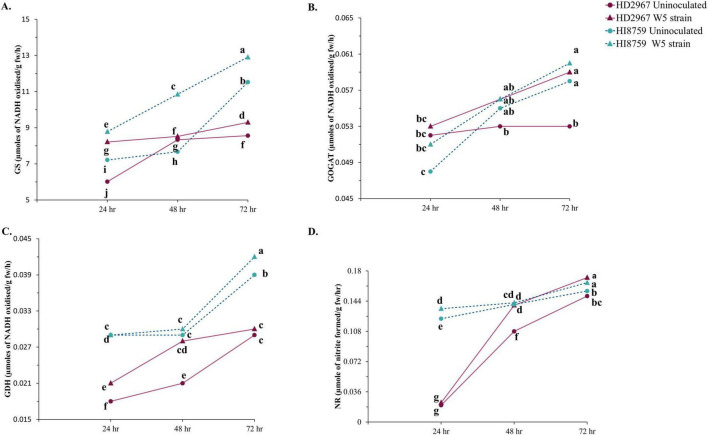
Temporal changes in the N assimilating enzymatic activities in seedlings on 24, 48, and 72 h due to inoculation with W5 strain for two wheat genotypes, HD2967 & HI8759. **(A)** Glutamine synthetase. **(B)** Glutamate synthase. **(C)** Glutamate dehydrogenase. **(D)** Nitrate reductase. Different lowercase letters on error bars indicate statistically significant differences between the treatments (*p* ≤ 0.05) performed by the Least significant difference test.

The influence of *A. chroococcum* W5 on N-assimilating enzyme activities in the seedling was determined for 72 h. The beneficial effect of inoculation on GS and GOGAT enzyme activities was observed for both wheat varieties, with higher activity in inoculated treatments (Figures 5A,B). For GS activity, both the inoculated varieties showed a significant increase compared to uninoculated, with the highest GS activity at 72 h for inoculated HI8759 (12.9 γ-glutamyl hydroxamate production/ g fw/ h). After 48 h, we did not observe a significant increase in GOGAT activity for both varieties compared to the uninoculated treatment. However, at 72 h, there was a significant increase of 11.32% in inoculated HD2967 (0.0533 μmoles NADH oxidized/g fw/h) compared to uninoculated HD2967 (0.059 μmoles NADH oxidized/g fw/h).

GDH enzyme activity increased over time, and treatments with *A. chroococcum* W5 show higher enzyme activity compared to their corresponding controls (Figure 5C). Inoculated HI8759 showed the highest activity (0.042 μmol NADH oxidized/g fw/h) at 72 h compared to uninoculated HI8759 (0.039 μmol NADH oxidized/g fw/h). NR enzyme activity also increased over time for all the treatments (Figure 5D). Highest activity was observed in the inoculated HD2967 (0.172 μmol nitrite formed/g fw/h) at 72 h compared to uninoculated HD2967 (0.166 μmol nitrite formed/g fw/h). Finally, the effect of *A. chroococcum* W5 on phytohormone GA content was also analyzed. We observed an increase of GA content in the seedlings for both wheat varieties in the presence of *A. chroococcum* W5 (Supplementary Figure 3).

### 3.6 The metabolic profile in *Azotobacter chroococcum* W5 reveals adaptative responses to abiotic stress

**FIGURE 6:**
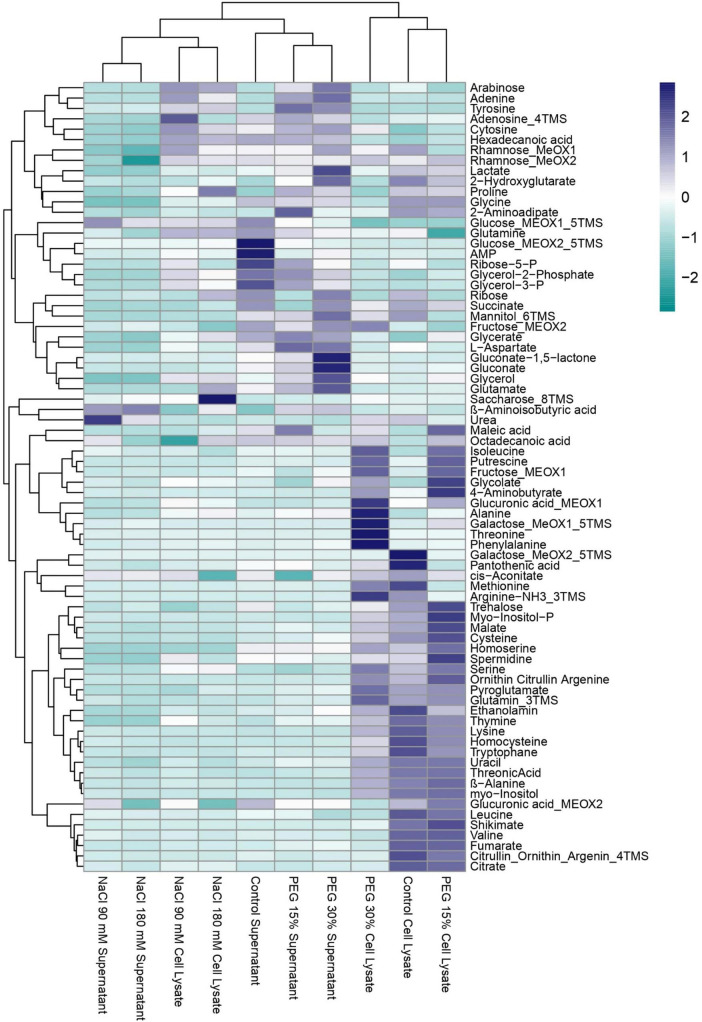
Metabolite Profile of *Azotobacter chroococcum* W5 with and without exposure to stress conditions (PEG 8000 (15 and 30%), salt (90 and 180 mM as NaCl). Detected metabolites include amino acids, organic acids, sugars, and other compounds, reflecting the metabolite decrease or increase in response to the different conditions.

We quantified both intra- and extracellular metabolites of *A. chroococcum* W5 in the presence of abiotic stress (90 mM and 180 mM salt concentration; 15 and 30% polyethylene glycol (PEG) representing −0.4425 and −1.027 Mpa, respectively) (Figure 6). Correlation analysis between metabolites in both cell lysates (Supplementary Figure 4) and supernatants (Supplementary Figure 5) were compared with bacterial growth. Several compounds, including lactate, urea, ethanolamine, and others, showed a strong positive correlation with growth in cell lysate under salt stress conditions. Conversely, compounds such as isoleucine, glutamate, and others exhibited a strong negative correlation with growth in the same conditions. In the supernatant, compounds like aminobutyric acid, proline, and others showed a strong negative correlation with growth. Conversely, compounds such as lactate, alanine, ethanolamine, and many others exhibited a strong positive correlation with growth. A list of all the metabolites in cell lysates and supernatants can be found in Supplementary Tables 7, 8.

Under osmotic stress conditions, treatments showed decreased levels of sugars and organic compounds compared to growth. For instance, metabolites from supernatants like lactate, alanine, and others exhibited a strong negative correlation with growth. Conversely, compounds such as glycerol and glucose showed a strong positive correlation with growth. In cell lysate under osmotic stress, compounds including lactate, valine, and others displayed a strong positive correlation with growth, whereas glycolate, alanine, and others showed a strong negative correlation (Supplementary Figures 4, 5).

Under salt stress conditions, metabolites change between cell lysates and supernatants. Proline increased significantly in both cell lysates and supernatants, with higher fold changes observed at 180 mM salt concentration. Glycerol, increased in cell lysate under salt stress. Conversely, trehalose levels decreased in cell lysate with increasing salt concentrations. Threonine decreased in the supernatant under salt stress. β-aminobutyric acid significantly increased in the supernatant under salt stress conditions. Under PEG-induced osmotic stress, proline decreased in cell lysates but increased in the supernatants, while 2-hydroxyglutarate levels in the supernatant significantly increased (Supplementary Figures 4, 5).

## 4 Discussion

Previous studies of *A. chroococcum* have primarily focused on identifying key genes involved in siderophore production (Zhang et al., 2019; Johnson et al., 2006), nitrogenase activity, and phosphorus mobilization (Robson et al., 1989; Sellamuthu et al., 2018; Biełło et al., 2023; Jain et al., 2021). Here, we conducted a comprehensive comparative genomic analysis of *A. chroococcum* W5 and related *Azotobacter* species, revealing unique genetic features potentially involved in plant growth promotion activities and specialized metabolite production. By incorporating tools like PLaBAse into our research, we identified around 1,643 functional traits that were associated with genes encoded in the W5 strain. These findings were complemented with phenotypic assays in wheat, which confirmed the W5 strain’s ability to enhance seedling vigor, produce phytohormones, as well as its capacity for nitrogen fixation and phosphate solubilization.

**FIGURE 7:**
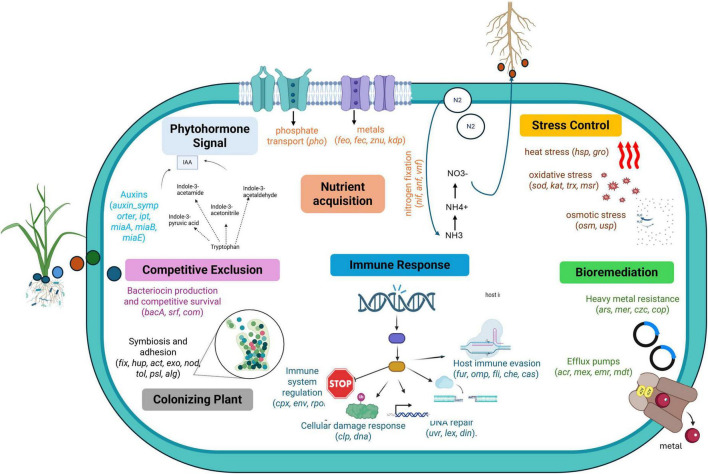
Schematic overview of the principal plant growth promotion genes of W5 strain predicted by PLaBAse platform. The genes are colored according to direct effects (nutrient acquisition, orange; phytohormone plant signal production, light blue; bioremediation, green) and indirect effects (stress control, yellow; competitive exclusion, pink; immune response dark blue; colonizing plant, gray). Complete gene names and descriptions are provided in Supplementary Table 1.

Although PLaBAse is a valuable resource for relating genes to plant growth-promoting traits (PGPTs) used in recent studies (Ayala Zepeda et al., 2024), it is important to recognize that predictions are highly dependent on gene annotations and sequence similarity. These factors may not reflect actual gene expression or functionality under specific environmental conditions. Additionally, broad trait categories (like stress tolerance or competitive exclusion) can include genes that are primarily associated with microbial survival rather than providing direct benefits to plants. This could result in an overestimation of the observed effects. Nevertheless, PLaBAse can serve as a useful tool for annotating and identifying genes as a first exploration, integrating this knowledge into phenotypic validation (Figure 7).

Our pan-genome analysis revealed a higher number of core, accessory, and unique genes than previously reported (Abdul Aziz et al., 2024), with approximately 88% classified as accessory genes. Although most unique genes were not classified in a KEGG category, many were related to DNA repair, and recombination processes, mainly for maintaining cellular integrity and responding to environmental stress (Du et al., 2022). For instance, the detection of pilD and tadE/tadG genes in *A. chroococcum* W5 suggests a potential role in bacterial adhesion and biofilm formation. pilD encodes a prepilin peptidase essential for the maturation of type IV pilins (Linhartová et al., 2021), while tadE and tadG are components of the Tad (tight adherence) secretion system, known to be involved in the assembly of adhesive pili (Ruiz-Muñoz et al., 2025)

Biosynthetic gene cluster analysis identified 115 BGCs in *Azotobacter* genomes, consistent with previously reported analysis (Johnson et al., 2006), where NRP-metallophores and NI-siderophores (17.15%) were present in all strains and have been studied for modulating plant protection (Biełło et al., 2023). *A. chroococcum* W5 exhibited one of the highest numbers of BGCs, and its antimicrobial and antifungal potential (Tarana et al., 2024; Bhosale et al., 2013) has been demonstrated with inhibition assays. According to homology networks, approximately 58% of all BGCs in *Azotobacter* networks lack classification according to the MiBIG database, representing a significant unexplored functional diversity database. This uncharacterized portion of the biosynthetic repertoire holds considerable biotechnological potential, as it may harbor novel metabolites with antimicrobial, signaling, or growth-promoting functions (Baars et al., 2017). For example, previous genetic interventions in *Azotobacter*, such as nifL knockout to increase ammonium release (Ortiz-Marquez et al., 2012; Bageshwar et al., 2017), and transgenic expression of glucose dehydrogenase to enhance phosphate solubilization (Sashidhar and Podile, 2009), demonstrate the strain’s utility in agricultural biotechnology. Our genome analysis identified promising targets for similar optimization in the W5 strain (Galindo et al., 2007).

Experimental assays confirmed that strain W5 produces auxin and gibberellic acid, solubilizes phosphate, fixes nitrogen moderately, and can utilize ACC as a nitrogen source, thereby supporting its plant growth-promoting (PGP) potential. In seed germination assays with wheat, inoculation with W5 significantly enhanced radicle and plumule length as well as seedling vigor, particularly when live cells were applied. The presence of metabolically active cells likely ensured a sustained release of phytohormones and other beneficial metabolites, contributing to the superior performance observed in treatments with live inoculation. These findings are consistent with previous reports on *Azotobacter vinelandii* and other rhizobacteria, where bacterial inoculation has been shown to improve germination and early seedling development (Golkar, 2019; Smirnova et al., 2024; Hura, 2020). The elevated GA content in inoculated seedlings contributed to higher enzymatic activities that enhance nutrient mobilization (Taiz et al., 2015), explaining the increased starch breakdown and free sugar accumulation observed. In the same way, W5 strain inoculation enhanced the activity of nitrogen assimilation enzymes (GS, GOGAT, GDH, NR), similar to findings in cotton and mustard plants (Romero-Perdomo et al., 2017; Lavanya et al., 2024). In contexts of limited nitrogen fertilization, such enhancement of assimilation enzymes becomes even more relevant, offering a promising strategy to improve crop productivity while reducing dependency on synthetic fertilizers (Mokhele et al., 2012).

Our analysis revealed a significant correlation between amino acids and intermediate metabolites, potentially linked to plants’ phytohormone production and stress-protective mechanisms. The metabolomic analysis of bacterial supernatant revealed the presence of tryptophan, an essential precursor for IAA synthesis, indicating the presence of pathway for the biosynthesis of IAA, which was also confirmed experimentally. *A. chroococcum* has been noted to produce IAA in earlier reports also (Apte and Shende, 1981), thus corroborating present findings (Etesami and Glick, 2024).

Regarding tolerance to stress, we observed several changes in the metabolic profile of *A. chroococcum* W5 when exposed to osmotic stressors. There was a significantly high adverse effect on bacterial growth, with 48.5% and 74.5% growth in the presence of 15% and 30% PEG, respectively, along with a concurrent increase in the concentration of glycerol and putrescine in the cell lysate (Supplementary Figure 4). These metabolites have been previously associated with stress protection in bacteria (Schneider et al., 2013; Colina et al., 2020), suggesting their possible role in providing limited protection to *A. chroococcum* W5 under osmotic stress. However, a positive correlation between growth, proline, 2-hydroxyglutarate, glycine, and AMP was noted, suggesting a significant decrease in their concentration in the osmotically stressed cells. There was higher concentration of the osmolytes proline, glutamate and glycerol, and signaling metabolites 2-hydroxyglutarate, in the supernatants of the treatments with high level of PEG and these showed negative correlation with bacterial growth (Supplementary Figure 5), suggesting that osmotic stress possibly disrupted cellular functions related to energy production, membrane stability, leading to their secretion from the injured cells. Negative correlation of bacterial growth in cell lysate with threonine, which is essential for cell membrane integrity, also indicated possible cell membrane injury, resulting in reduced growth and leakage of cellular constituents.

In summary, our findings demonstrated the strong potential of *Azotobacter chroococcum* W5 to promote plant growth. Functional assays confirmed the bacterium’s abilities to produce phytohormones, fix nitrogen, and solubilize phosphate. These factors are associated with enhanced wheat germination and seedling vigor, although functional validation through gene knockout or mutational analysis is required to confirm causality. Additionally, regulating both primary and secondary metabolite production is crucial for wheat seed germination and the synthesis of antimicrobial compounds involved in plant defense. This study lays the groundwork for future investigations into the generation of formulations with *Azotobacter* strains for innovative agricultural applications.

## Data Availability

The genome sequences analyzed in this study are publicly available in the NCBI repository, with the accession numbers listed in Table 1.
