# Supplementary material for: Comparative genomics and metabolomics reveal phytohormone production, nutrient acquisition, and osmotic stress tolerance in Azotobacter chroococcum W5
Source: Front Microbiol. 2025 Jul 22;16:1626016. doi: 10.3389/fmicb.2025.1626016 (PMC12322734; doi:10.3389/fmicb.2025.1626016)
Supplement: Supplementary file 2 [file Table_2.docx]

**Additional Table 2**. Pangenome statistics for *Azotobacter* genomes using BPGA.

| **Organism name** | **No. of core genes** | **No. of accessory genes** | **No. of unique genes** | **No. of exclusively absent genes** |
| --- | --- | --- | --- | --- |
| *Azotobacter beijerinckii DSM 282* | 2410 | 1828 | 118 | 5 |
| *Azotobacter beijerinckii DSM 373* | 2410 | 1842 | 183 | 0 |
| *Azotobacter beijerinckii DSM 378* | 2410 | 1703 | 172 | 6 |
| *Azotobacter beijerinckii DSM 381* | 2410 | 1832 | 88 | 0 |
| *Azotobacter beijerinckii DSM 1041* | 2410 | 1805 | 227 | 6 |
| *Azotobacter chroococcum ATCC 9043* | 2410 | 1803 | 44 | 5 |
| *Azotobacter chroococcum B3* | 2410 | 1562 | 108 | 35 |
| *Azotobacter chroococcum DSM 2286* | 2410 | 1849 | 77 | 1 |
| *Azotobacter chroococcum HR1* | 2410 | 1700 | 183 | 3 |
| *Azotobacter chroococcum isscasi P205* | 2410 | 1480 | 112 | 3 |
| *Azotobacter chroococcum NCIMB 8003* | 2410 | 1589 | 121 | 5 |
| *Azotobacter chroococcum P204* | 2410 | 1710 | 115 | 3 |
| *Azotobacter chroococcum P207* | 2410 | 1450 | 144 | 4 |
| *Azotobacter chroococcum P208* | 2410 | 1764 | 252 | 5 |
| *Azotobacter chroococcum W5* | 2410 | 1530 | 112 | 2 |
| *Azotobacter salinestris KACC 13899* | 2410 | 1551 | 458 | 43 |
| *Azotobacter vinelandii CA* | 2410 | 2393 | 1 | 1 |
| *Azotobacter vinelandii CA6* | 2410 | 2356 | 0 | 21 |
| *Azotobacter vinelandii DJ* | 2410 | 2398 | 2 | 1 |
| *Azotobacter vinelandii DSM 279* | 2410 | 2135 | 228 | 2 |
| *Azotobacter vinelandii NBRC 13581* | 2410 | 1912 | 234 | 2 |
| *Azotobacter vinelandii VKMB-1617* | 2410 | 2071 | 85 | 12 |
