# Supplementary material for: Comparative genomics and metabolomics reveal phytohormone production, nutrient acquisition, and osmotic stress tolerance in Azotobacter chroococcum W5
Source: Front Microbiol. 2025 Jul 22;16:1626016. doi: 10.3389/fmicb.2025.1626016 (PMC12322734; doi:10.3389/fmicb.2025.1626016)
Supplement: Supplementary file 5 [file Table_5.docx]

**Additional Table 5**. Antismash results for *A. chroococcum* W5.

| Region | Putative type | From | To | Most similar known cluster | Similarity |
| --- | --- | --- | --- | --- | --- |
| Region 1.1 | NI-siderophore | 279,176 | 293,841 | vibrioferrin | High |
| Region 6.1 | NRPS | 15,058 | 59,038 |  |  |
| Region 12.1 | T3PKS,hglE-KS | 343,233 | 393,288 |  |  |
| Region 23.1 | redox-cofactor | 337,909 | 360,169 |  |  |
| Region 23.2 | betalactone | 362,876 | 386,726 |  |  |
| Region 32.1 | NRPS,NRP-metallophore,T1PKS | 61,919 | 139,288 | crochelin A | High |
| Region 32.2 | RiPP-like | 264,212 | 276,416 |  |  |
| Region 34.1 | NAGGN | 152,227 | 166,998 |  |  |
| Region 35.1 | phenazine | 86,531 | 106,956 |  |  |
| Region 35.2 | betalactone | 222,361 | 252,745 |  |  |
