# Supplementary material for: Comparative genomics and metabolomics reveal phytohormone production, nutrient acquisition, and osmotic stress tolerance in Azotobacter chroococcum W5
Source: Front Microbiol. 2025 Jul 22;16:1626016. doi: 10.3389/fmicb.2025.1626016 (PMC12322734; doi:10.3389/fmicb.2025.1626016)
Supplement: Supplementary file 6 [file Table_6.docx]

**Additional Table 6**. A-B. Changes in the N assimilating enzymatic activities and free-sugar assimilation in the seedling on 24h, 48h and 72h due to inoculation of W5 strain for 2 wheat genotypes, HD2967 & HI8759. C. GA content on the 3rd day of bacterial inoculation.

| Parameters |  | Total starch concentration % | | | | Amylose concentration % | | | |  | Amylopectin concentration % | | | |
| --- | --- | --- | --- | --- | --- | --- | --- | --- | --- | --- | --- | --- | --- | --- |
| Day | Treatment | HD 2967 | Std | HI 8759 | Std | HD 2967 | Std | HI 8759 | Std |  | HD 2967 | Std | HI 8759 | Std |
| **1** | **Uninoculated** | 74.220^c^ | 0.502 | 89.955^a^ | 0.781 | 16.716^e^ | 0.046 | 32.996^a^ | 0.046 |  | 83.284^f^ | 0.046 | 67.004^k^ | 0.046 |
|  | **W5 strain** | 81.285^b^ | 0.852 | 74.040^c^ | 2.912 | 11.763^g^ | 0.000 | 22.801^c^ | 0.436 |  | 88.237^d^ | 0.000 | 77.199^h^ | 0.436 |
| **2** | **Uninoculated** | 60.960^e^ | 0.350 | 58.290^f^ | 0.332 | 15.610^f^ | 0.046 | 26.805^b^ | 0.091 |  | 84.390^e^ | 0.046 | 73.195^j^ | 0.091 |
|  | **W5 strain** | 64.500^d^ | 0.793 | 37.950^j^ | 1.498 | 11.236^h^ | 0.046 | 20.641^d^ | 0.121 |  | 88.764^c^ | 0.046 | 79.359^g^ | 0.121 |
| **3** | **Uninoculated** | 52.202^h^ | 1.216 | 54.795^g^ | 0.542 | 10.236^i^ | 0.319 | 23.012^c^ | 0.046 |  | 89.580^b^ | 0.000 | 76.961^i^ | 0.046 |
|  | **W5 strain** | 47.340^i^ | 0.234 | 26.505^k^ | 1.768 | 9.603^j^ | 0.255 | 16.900^e^ | 0.000 |  | 90.265^a^ | 0.046 | 83.100^f^ | 0.000 |

B.

| Parameters |  | GS (µmoles of NADH oxidised/g fw/h) | | | |  | GOGAT (µmoles of NADH oxidised/g fw/h) | | | |  | GDH (µmoles of NADH oxidised/g fw/h) | | | |  | NR (µmole of nitrite formed/g fw/hr) | | | | |
| --- | --- | --- | --- | --- | --- | --- | --- | --- | --- | --- | --- | --- | --- | --- | --- | --- | --- | --- | --- | --- | --- |
| Day | Treatment | HD 2967 | Std | HI 8759 | Std |  | HD 2967 | Std | HI 8759 | Std |  | HD 2967 | Std | HI 8759 | Std |  | HD 2967 | Std | HI 8759 | Std |  |
| **1** | **Uninoculated** | 6.009^j^ | 0.109 | 7.215^i^ | 0.109 |  | 0.052^bc^ | 0.002 | 0.048^c^ | 0.004 |  | 0.018^f^ | 0.001 | 0.029^d^ | 0.003 |  | 0.020^g^ | 0.005 | 0.123^e^ | 0.002 |  |
|  | **W5 strain** | 8.210^g^ | 0.040 | 8.778^e^ | 0.026 |  | 0.053^bc^ | 0.006 | 0.051^bc^ | 0.003 |  | 0.021^e^ | 0.002 | 0.029^c^ | 0.003 |  | 0.023^g^ | 0.003 | 0.135^d^ | 0.002 |  |
| **2** | **Uninoculated** | 8.332^g^ | 0.027 | 7.668^h^ | 0.015 |  | 0.053^b^ | 0.002 | 0.055^ab^ | 0.003 |  | 0.021^e^ | 0.001 | 0.029^c^ | 0.002 |  | 0.108^f^ | 0.002 | 0.140^d^ | 0.002 |  |
|  | **W5 strain** | 8.516^f^ | 0.026 | 10.848^c^ | 0.026 |  | 0.056^ab^ | 0.002 | 0.056^ab^ | 0.002 |  | 0.028^cd^ | 0.002 | 0.030^c^ | 0.001 |  | 0.139^d^ | 0.004 | 0.142^cd^ | 0.004 |  |
| **3** | **Uninoculated** | 8.559^f^ | 0.015 | 11.521^b^ | 0.055 |  | 0.053^b^ | 0.003 | 0.058^a^ | 0.003 |  | 0.029^c^ | 0.002 | 0.039^b^ | 0.002 |  | 0.150^bc^ | 0.003 | 0.156^b^ | 0.012 |  |
|  | **W5 strain** | 9.293^d^ | 0.129 | 12.909^a^ | 0.304 |  | 0.059^a^ | 0.002 | 0.060^a^ | 0.002 |  | 0.030^c^ | 0.000 | 0.042^a^ | 0.001 |  | 0.172^a^ | 0.011 | 0.166^a^ | 0.004 |  |

C.

|  | GA (µg/g FW) | | | |
| --- | --- | --- | --- | --- |
| Treatment | HD 2967 | Std | HI 8759 | Std |
| **Uninoculated** | 0.629^c^ | 0.041 | 0.538^d^ | 0.004 |
| **W5 strain** | 0.813^a^ | 0.000 | 0.733^b^ | 0.001 |
