# Supplementary figures and images for: Comparative genomics and metabolomics reveal phytohormone production, nutrient acquisition, and osmotic stress tolerance in Azotobacter chroococcum W5
Source: Front Microbiol. 2025 Jul 22;16:1626016. doi: 10.3389/fmicb.2025.1626016 (PMC12322734; doi:10.3389/fmicb.2025.1626016)

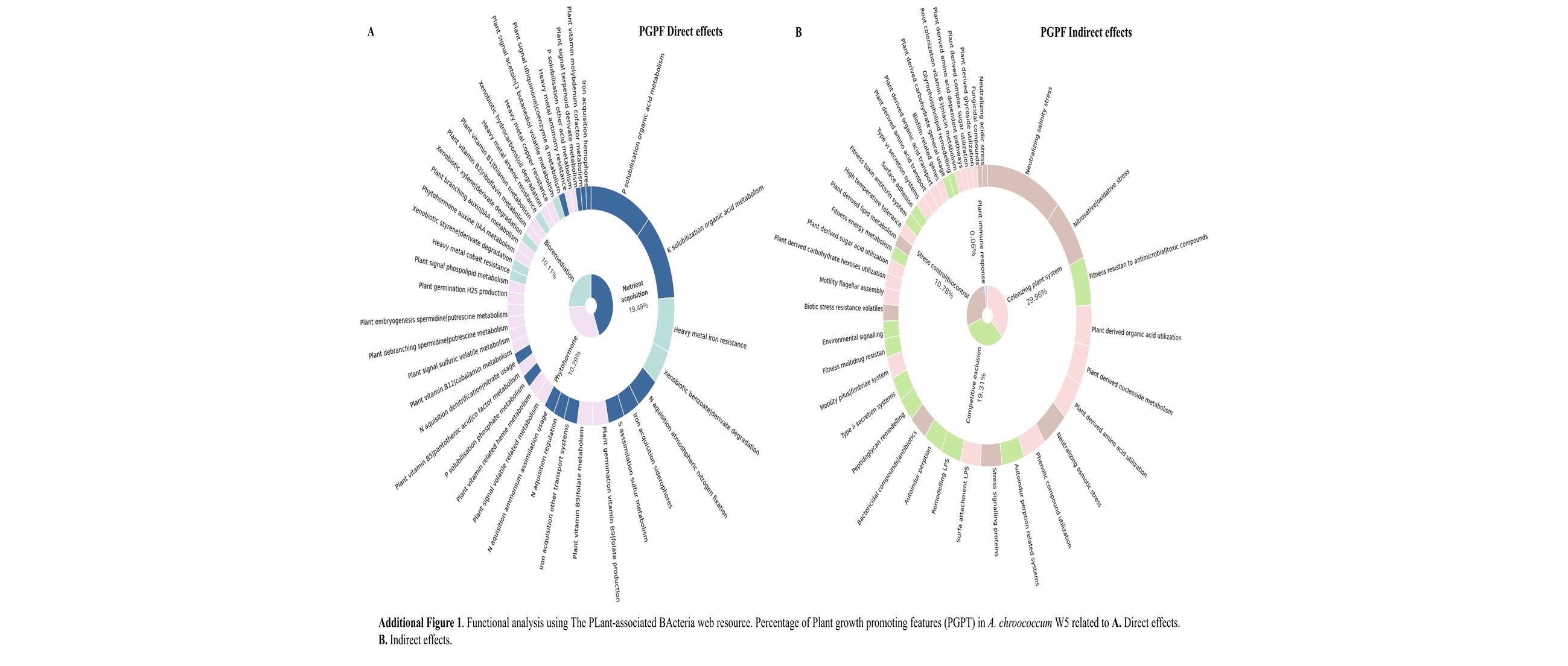

Supplement: Supplementary file 9 [file Image_1.jpeg]

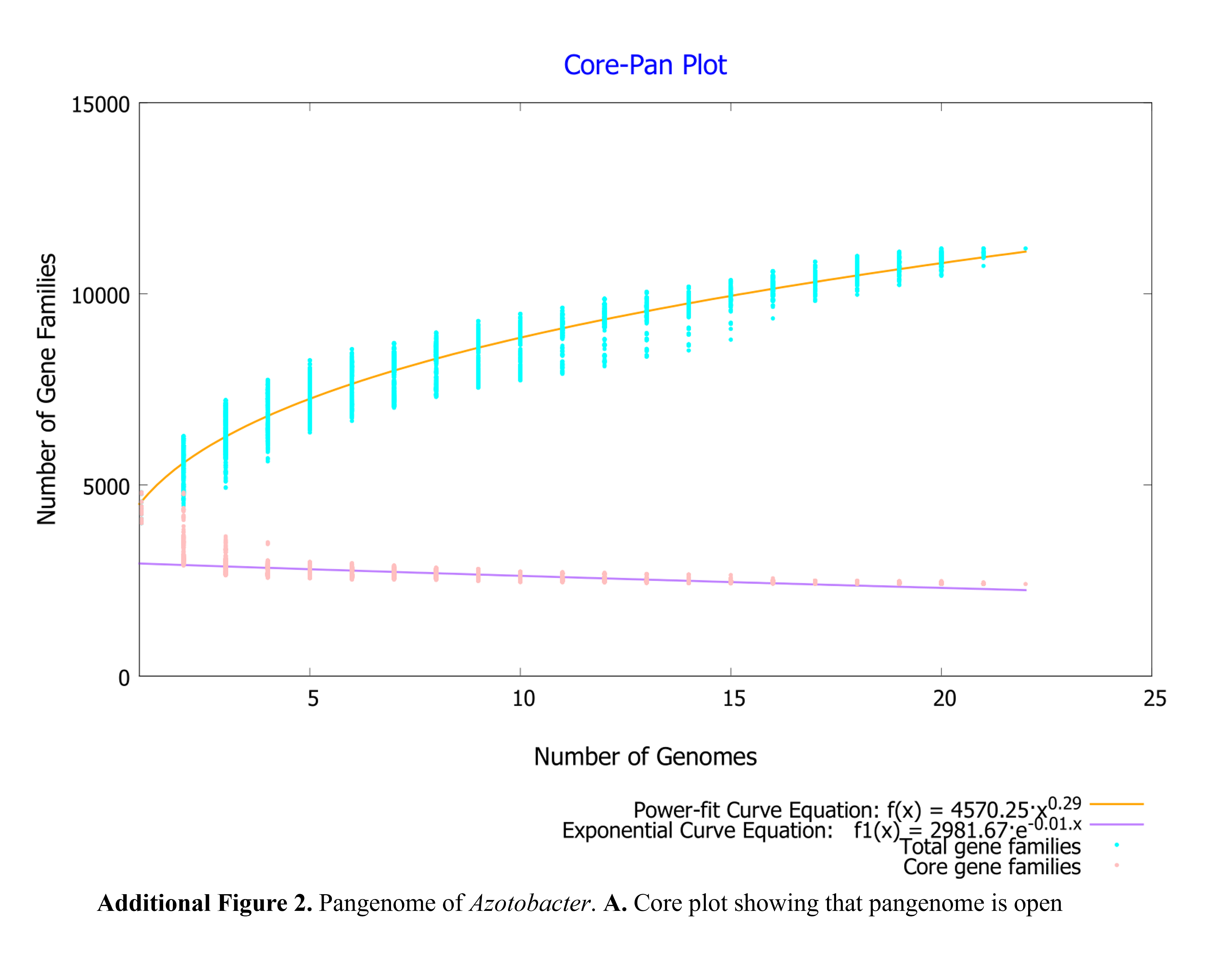

Supplement: Supplementary file 10 [file Image_2.tif]

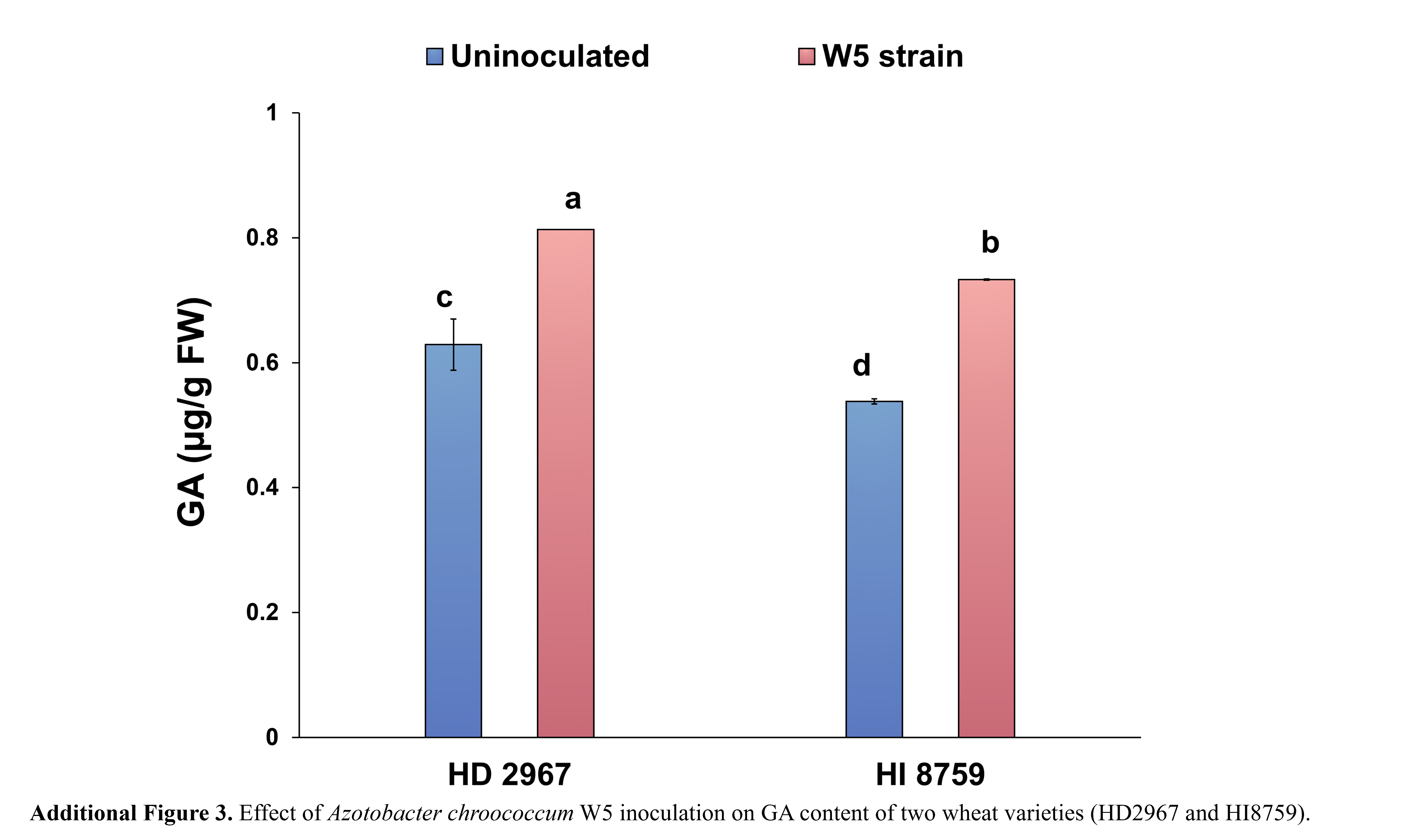

Supplement: Supplementary file 11 [file Image_3.tif]

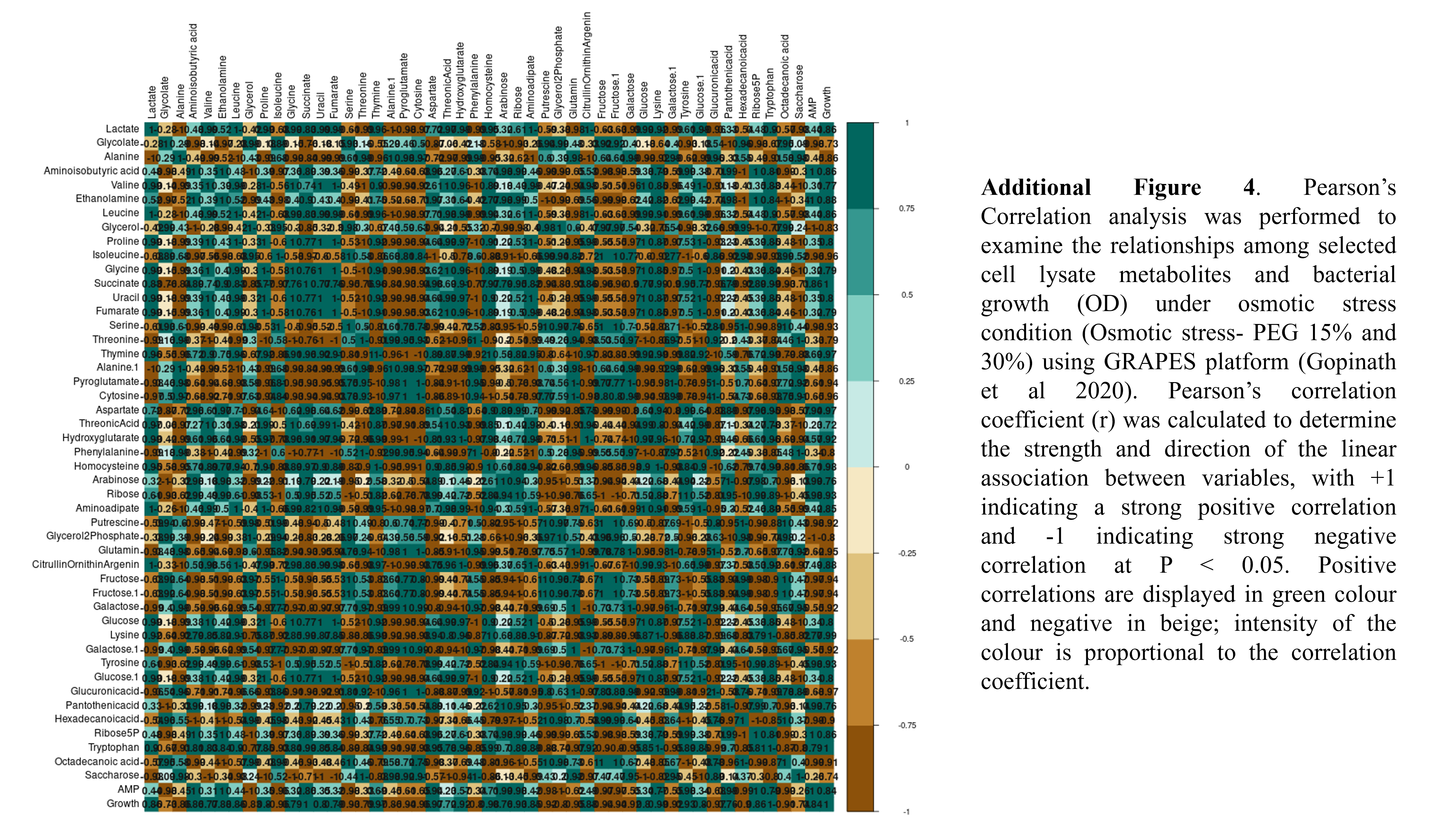

Supplement: Supplementary file 12 [file Image_4.tif]

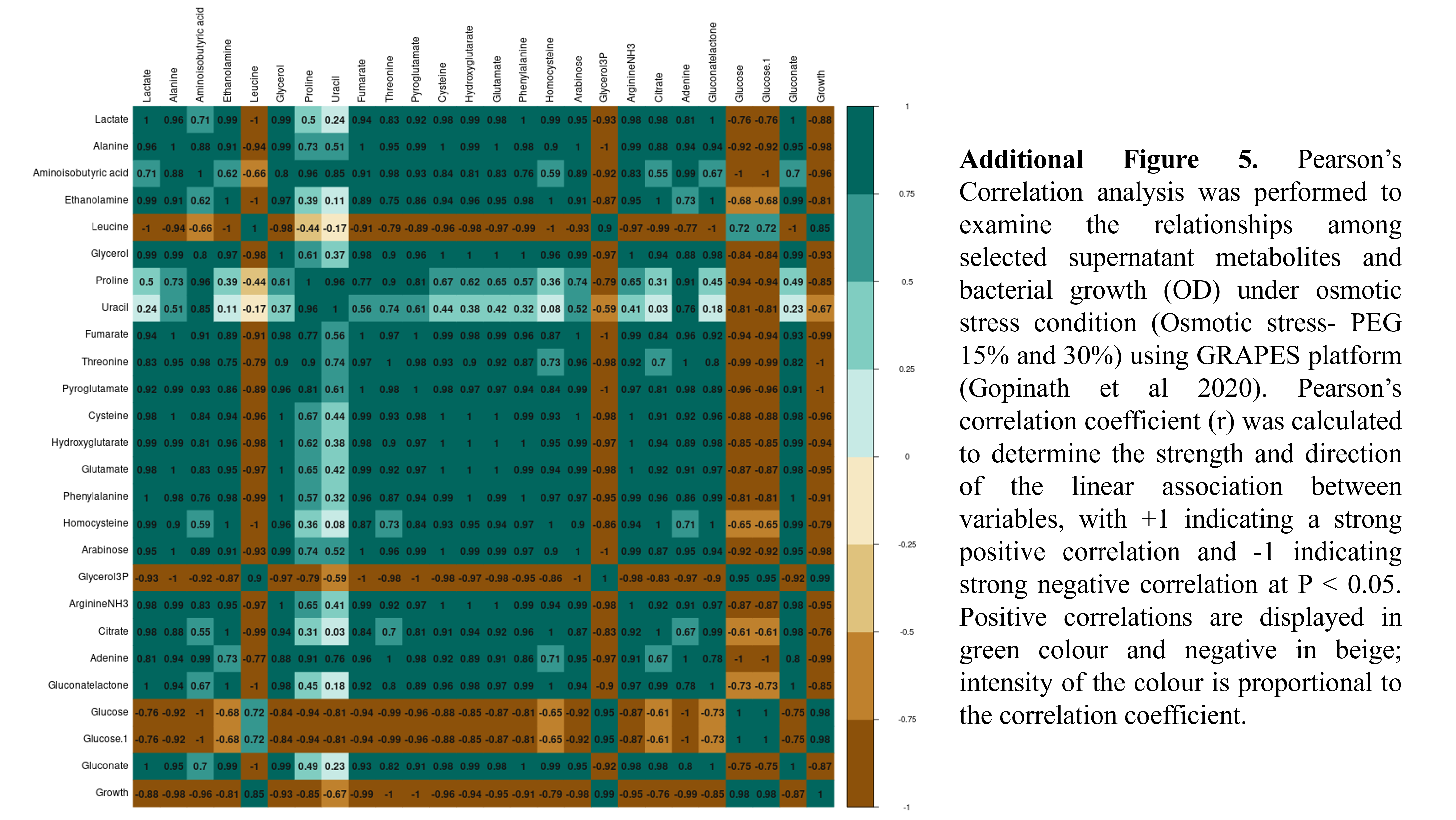

Supplement: Supplementary file 13 [file Image_5.tif]
